# Reference Values of Forced Vital Capacity and Expiratory Flow in High-Level Cyclists

**DOI:** 10.3390/life11121293

**Published:** 2021-11-25

**Authors:** Marc Dauty, Thomas Georges, Camille Le Blanc, Bastien Louguet, Pierre Menu, Alban Fouasson-Chailloux

**Affiliations:** 1 CHU Nantes, Service de Médecine Physique et Réadapatation Locomotrice et Respiratoire, 44093 Nantes, France; marc.dauty@chu-nantes.fr (M.D.); thomas.georges@chu-nantes.fr (T.G.); camille.leblanc@chu-nantes.fr (C.L.B.); pierre.menu@chu-nantes.fr (P.M.); 2 CHU Nantes, Service de Médecine du Sport, 44093 Nantes, France; bastien.louguet@chu-nantes.fr; 3 IRMS—Institut Régional de Médecine du Sport, 44093 Nantes, France; 4 Inserm, UMR 1229, RMeS—Regenerative Medicine and Skeleton, Université de Nantes, ONIRIS, 44093 Nantes, France

**Keywords:** sport, cycling, lung volume, spirometry, anthropometry

## Abstract

Several studies have demonstrated that spirometric theoretical values may not be applicable to the high-level sports population. No reference values exist for high-level professional cyclists. We aimed to establish predictive spirometric values by reference equations. One hundred and forty-five French Caucasian high-level professional cyclists, aged 18–38, performed basic anthropometric assessment and spirometry during the medical evaluation at the beginning of the sport season. Measured values were compared with theoretical values. Predictive equations were established from anthropometric parameters to explain variations of spirometric parameters. High-level cyclists had significantly higher spirometric values than the theoretical values established from a general population, except for forced expiratory volume in one second (FEV_1_), forced vital capacity (FVC) and forced expiratory flow (FEF) at 25% of FVC. Only FVC and FEV_1_ were well predicted from body height. The FVC variation of 43.5% is explained by body height and weight. The FEV_1_ variation of 25.8% is explained only by body height. High-level cycling is associated with important respiratory adaptations depending on the body height and the sport specificity: intensive and prolonged endurance training. These findings are interesting for clinical individual application to diagnose obstructive disease and test reversibility with bronchodilator drugs.

## 1. Introduction

During spirometry, air volumes and flows are expressed in absolute values and as percentages of the predicted or theoretical values (Th) [1]. These theoretical values have been established from cohorts of healthy adults with the same anthropometric characteristics (sex, age from 18 to 70 and height from 155 to 195 cm for men) [2,3,4]. If the absolute value is less than 80% or more than 120% of the theoretical value, it is considered abnormal [5].

In high-level athletes, spirometry is performed either before a conventional graded exercise or when there is asthma, allergy or symptoms suggestive of exercise-induced bronchoconstriction [6,7,8]. An obstructive ventilatory disorder is then sought and diagnosed when the forced expiratory volume in one second (FEV_1_) is under 80%Th and the FEV_1_/FVC (forced vital capacity) is under 70%Th or the FEV_1_/FVC ratio is below the 5th percentile of the theoretical value [5]. The volume flow curve corresponds to a concave curve by decreasing the values of the forced expiratory flow at 75, 50 and 25% of the FVC (FEF75, FEF50 and FEF25) according to a value of maximal midexpiratory flow (MMEF) 25–75 < 50%Th [5]. This obstructive disorder is considered reversible if short-acting bronchodilator therapy improves FEV_1_ by 200 mL and 12% over the original value. In this case, bronchodilator therapy can then be used in competition according to the recommendations of the International Olympic Committee and the limits imposed by the Anti-Doping Agency [8,9]. High-level endurance athletes, especially professional cyclists, can present exercise-induced bronchoconstriction during their career due to asthma or allergy in 50 to 90% of cases [8,10]. The prevalence of exercise-induced bronchoconstriction without asthma could be 10 to 50% in high-level athletes, perhaps linked with specific environmental pollution or/and hyperventilation [8]. Furthermore, lung volume is significantly higher than predicted values in high-level athletes [11]. However, the exact influence of the sport level and the anthropometric parameters are not well known because body height represents a major confounding factor. Strong positive correlations were shown with spirometric values in basketball and water polo players [11]. Moreover, a distinction of the type of high-level sports seems to be interesting because of specific differences between power and endurance sports [12]. The prolonged demand for gas exchange, which increases the strength of respiratory muscles, could explain high spirometric values in endurance athletes [12]. All these arguments explain why predicted spirometric values established from a general, non-athletic population should not be taken into account in high-level athletes [13]. Athletes’ normal lung volumes may be underestimated and this could mask obstructive lung diseases which may be detected after bronchial dilatation tests [10,14].

Few data exist in high-level cyclists who practice endurance sport with a potential to develop obstructive diseases. The objective of this study was to describe spirometric values and establish reference equations in this specific sport population who have similar body height to the general population.

## 2. Materials and Methods

### 2.1. Participants and Procedures

All adult Caucasian high-level professional road cyclists (UCI WorldTeam or Continental ProTeam) were included at the beginning of the sport season from 2014 to 2019 during the medical evaluation imposed by the French Cycling Federation and the UCI World Cycling to obtain a professional cycling license. Subjects who had had a recent respiratory infection or taken any pulmonary medications in the last 3 weeks were not included. High-level professional cyclists with a history of pulmonary diseases such as asthma, bronchoconstriction induced by exercise or allergy were excluded.

### 2.2. Measures

The anthropometric assessment included body height to the nearest 0.1 cm (wall height rod Seca 222^®^, Semur-en-Auxois, France), bodyweight to the nearest 0.1 kg (Scale Seca 760 Colorata^®^, Semur-en-Auxois, France). The body mass index was calculated according to the formula: weight/height^2^ (kg/m^2^). 

The pulmonary evaluation was performed according to the guidelines of the American Thoracic Society/European Respiratory Society Task Force (ATS/ERS) [15]. Sportsmen were instructed not to exercise, to drink alcohol, caffeine or theophylline beverages prior to the spirometry tests. All the tests were performed with the same Pony FX^®^ spirometer (Cosmed^®^, Brignais, France) in the morning (between 9:00 and 11:00 a.m.). Measurements were carried out under a comfortable temperature (18–22 °C), at a pressure of 760 mmHg and relative humidity between 30 and 60%. The temperature, atmospheric pressure and humidity were continuously monitored to keep with the body temperature and pressure, saturated (BTPS) conditions. The volume signal was calibrated daily with a 3.0 L syringe according to the manufacturer’s recommendations [16]. Measured and theoretical values were automatically recorded by the computer. The theoretical values were those of the ERS TASK FORCE Global Lung Initiative 2012 [4].

The procedure was conducted in the sitting position while the sportsmen used a nose clip. The sportsmen were then instructed to use a mouthpiece and to follow the procedure: (1) breathe quietly and normally, (2) inspire completely and rapidly with a pause ≤2 s, (3) expire with maximal effort for at least 6 s, until no more air could be expelled while maintaining an upright posture and, (4) inspire with maximal effort until completely full [15]. Two or three acceptable trials were required for each cyclist to check FEV_1_ and FVC repeatability (≤0.150 L) and to record the best values (grade A and B of the spirometry quality grading system) [17]. FVC, FEV_1_, FEV_1_/FVC, peak expiratory flow (PEF), FEF at 75, 50, 25% of FVC and maximal midexpiratory flow between 25 and 75% of FVC (MMEF 25–75) were taken into consideration [15].

### 2.3. Statistical Analysis

Statistical analysis was performed with SPSS 23.0^®^ software (Armonk, NY, USA). Quantitative parameters were presented as mean, median, minimum, maximum and standard deviation (SD) or percentile [5,18]. The normality of data was tested by the Shapiro-Wilk test [19]. To compare the spirometric parameters of each subject to its theoretical values, we have transformed theoretical values (expressed in %) into continued variables. A paired Student’s *t*-test was used to compare measured and theoretical values. To build the flow-volume curve, we have used medians and the 5th and 95th percentiles. These parameters were also compared to theoretical values using a Wilcoxon rank test. The significant difference was determined at *p* < 0.05.

Stepwise multiple linear regressions were performed to identify the best predictions of lung function parameters in cyclists including lung volume as a dependent variable and anthropometric data (age, weight, height) as independent variables. A variable was added to the model if its associated *p* value for the *F* test was less than 0.05 and removed if its *p* value was greater than 0.1. The linear regression equation was then established from the partial coefficient *B* and the constant after checking different assumptions to valid results: (1) the independence of observations were tested using the Durbin-Watson test (acceptable for values between 1 and 3); (2) a linear relationship exists between dependent and independent variables; (3) verification of the homoscedasticity of variances; (4) absence of multicollinearity inspected by the correlation coefficients and tolerance/VIF values (<10); (5) absence of significant outliers after visualization of scatterplot and measure of influence according to Cook’s Distance [20]; (6) approximately normal distribution of studentized residuals (errors) explored by histogram *P-P* Plot.

The correlations were assessed with a correlation coefficient r and the r^2^ and r^2^_adj_ values were performed to indicate how much of the total variation in the dependent variable can be explained by the independent variable [21].

## 3. Results

### 3.1. Demographic Data

One hundred ninety-six high-level professional cyclists were eligible, but 51 of them (26%) were excluded for asthma or bronchoconstriction induced by exercise or allergy. Finally, 145 high-level professional cyclists were included. Their age was 23.7 ± 4.6 years, ranging from 18 to 38, their weight was 71.0 ± 6.9 kg, ranging from 59 to 102 kg, their height was 178.9 ± 6.0 cm, ranging from 168 to 197 cm and their body mass index (BMI) was 22.1 ± 1.6 kg/m^2^ ranging from 18.0 to 26.3. None of them were smokers.

### 3.2. Spirometric Data

**Figure 1:**
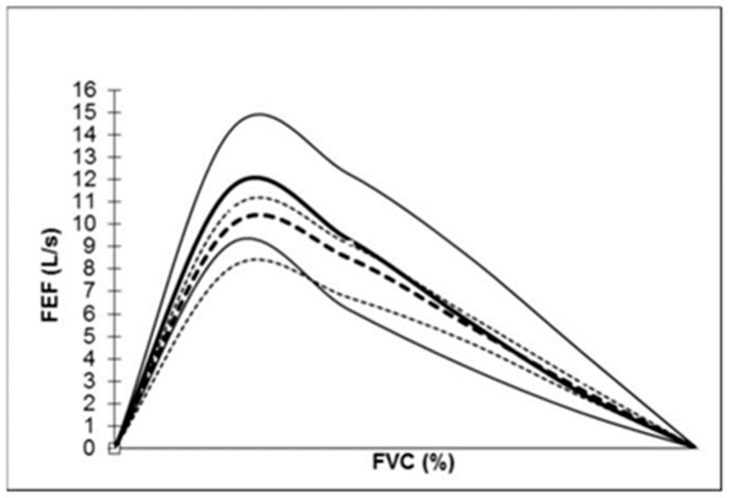
New flow-volume curve in the high-level professional cyclist population and theoretical curve in the general population. The solid line represents the cyclist flow-volume curve (median, 5th and 95th percentile). The dotted line represents the theoretical flow-volume curve (median 5th and 95th percentile). Abbreviations: FVC: forced vital capacity; FEF: forced expiratory flow.

The lung functional data are shown in Table 1 and the flow-volume curve of the cyclist population is presented with the median and the 10th and 90th percentile in Figure 1 with the comparison to the theoretical flow-volume curve. The mean of FVC, FEV_1_ and PEF were significantly superior to the theoretical values (≈115%) (Table 1 and Table 2). The FEV_1_/FVC values were not different from the theoretical values (84.1 ± 5.9%; min: 69%–max: 96%). The FEF75, FEF50 and the MMEF 25–75 were significantly superior to the theoretical values while the FEF25 were not (Table 2).

**Figure 2:**
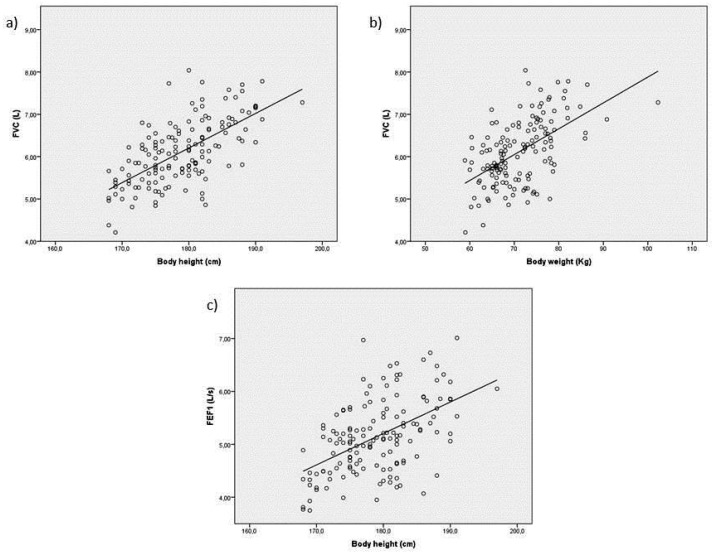
(**a**,**b**) Linear regression between FVC and body height (r = 0.644; *p* < 0.0001) and between FVC and body weight (r = 0.552; *p* < 0.0001); (**c**) linear regression between FEV_1_ and body height (r = 0.513; *p* < 0.0001).

Only FVC and FEV_1_ could be well predicted by linear regression equations: linear regressions between FVC and body height, and between FVC and bodyweight were r = 0.644 (*p* < 0.0001) and r = 0.552 (*p* < 0.0001), respectively; linear regression between FEV1 and body height was r = 0.513 (*p* < 0.0001) (Table 2 and Figure 2a–c). The FVC variation of 43.5% is explained by body height and weight. The FEV_1_ variation of 25.8% is explained only by body height. The variation of the other flows was not well predicted by age and anthropometric parameters.

## 4. Discussion

High-level professional cyclists have 15% higher FEV_1_, FVC and PEF spirometric values than theoretical values. Furthermore, FEF values at a percentage of FVC are higher than theoretical values, except for FEF25 (Figure 1). FEV_1_/FVC is in the general norm. There are few data available for high-level cyclists. Mazic et al., had reported the respiratory parameters of 39 cyclists aged 22.0 ± 4.0 years (weight 65.2 ± 7.5 kg; height 175.4 ± 6.6 cm; BMI: 21.1 ± 1.4 kg/m^2^) [14]. Absolute values of FEV_1_, FVC and PEF were lower than ours (FEV_1_: 4.49 ± 0.6 L/s; FVC: 5.13 ± 0.8 L and PEF: 9.14 ± 2.1 L). However, these values were higher than those of 16 sedentary subjects considered to be control subjects (FEV_1_: 107 ± 9%; FVC: 106 ± 9% and PEF: 102 ± 6%) [14]. The high professional sports level of our population probably accounted for the higher values even if the level of sport of the cyclist population of Mazic et al., was not mentioned [14]. Indeed, Medelli et al., had also reported similar spirometric measured values to ours (FEV_1_: 5.2 ± 0.7 L/s; FVC: 6.2 ± 0.9 L; PEF: 11.4 ± 1.7 L) in a cycling team of 25 high-level professional cyclists who presented similar anthropometric parameters as our population (height: 180 ± 6 cm and weight: 70 ± 7 kg) [10]. A significant difference between theoretical values predicted by the European Community for Coal and Steel and measured values of FVC, FEV_1_, PEF had already been shown [10]. In male athletes who practiced other endurance sports more than 15 h per week at a national or international level, FEV_1_, FVC, PEF values were higher than control sedentary subjects and also than theoretical values [11]. These findings could be of great importance in the diagnosis of respiratory disorders, especially in cases of airway obstruction [10,11]. In high-level athletes, endurance and aerobic training cause a significant improvement of PEF as compared to other types of training [13]. There is a cumulative effect of repeated sequences of endurance training, particularly during adolescence. Endurance high-level athletes (i.e., cycling, long distance running, triathlon) present significant high FVC and FEV_1_ compared to powerful high-level athletes (i.e., 100–200 m short distance running) or mixed discipline (i.e., soccer, basketball, handball, volleyball, tennis) [12]. In marathons, race time is correlated to the FVC/kg, FEV_1_/kg and PEF/kg (r = −0.400 to −0.500) and a high-level marathon group has a higher FEV_1_/kg and FVC/kg than other marathon groups of slower runners [22]. High ventilatory capacity is essential for sports such as cycling and is in relation with high maximum aerobic capacity (VO_2_), roughly linearly linked to minute ventilation (VE). During maximum exercise, most high-level cyclists reach a respiratory rate of around 55 breaths a minute [23]. Indeed, endurance exercise training demands high and prolonged ventilation. Inspiratory muscles can improve sport performance by reducing resistance in respiratory canals and increasing lung elasticity and alveolar expansion [12,24,25].

Anthropometric parameters such as body height and BMI also represent factors related to spirometry parameters in the general population, cyclists and high-level athletes [11,12,14]. Body height accounts for respiratory values in high-level sports subjects with strong positive correlations (r > 0.600 for FVC and FEV_1_ and >0.400 for PEF) [11,12]. Body height has also predicted FEV_1_ and FVC values directly in our high-level professional cyclist population. Compared to the results shown in cyclists by Mazic et al., the body height of our population was on average 3.5 cm higher and could explain our higher spirometric values [14]. Yet, body height did not explain all the lung parameters because for similar spirometric measures, our cyclists were smaller (<180 cm) than the top athletes studied by Lazovic et al., who practiced basketball (200 cm), water polo (191 cm), or rowing (193 cm) [12]. The FEV_1_, FVC and PEF values of our cyclists are rather explained by a high level of endurance practice. A genetic component can also be discussed because high-level cycling practice requires individual selection of athletes who possess outsized spirometry abilities in relation to their anthropometric parameters [26]. A difference in trunk length relative to standing height associated with fat-free mass, chest dimensions and strength of respiratory muscles may be genetically explained [5].

FEV_1_/FVC mean value corresponded to normal values and may be explained by the formulae which reported FEV_1_ to FVC, both parameters higher in the same proportion of 15% than theoretical values. This result was also shown in cyclists and high-level athlete populations [10,11,12,14]. The FEF at 75 and 50% of FVC was higher than theoretical values because the PEF was significantly higher, and no concave curve was present (Figure 1). Medelli et al., found the same results despite 2 of their 25 cyclists having asthma and seven a clinical history of allergy [10]. The FEF was not well predicted by anthropometric parameters. This result may be interesting to know for clinical applications to diagnose obstructive diseases and reversibility tests with bronchodilator drugs.

Our study presents some limits due to the relatively low number of 145 high-level professional cyclists to establish more robust spirometric reference equations. This is explained by the difficulty of recruiting cyclists practicing at such a sporting level. For instance, only 38 French high-level professional cyclists out of the 176 participants were engaged in the 2020 Tour de France. However, a number of 100 to 150 male subjects is sufficient to be confident that a significant difference between the published reference equations and the values from the local community does not exist [27]. The slow vital capacity (SVC) was not recorded during spirometric tests. This parameter would have been relevant in order to research a difference between SVC and FVC of more than 200 mL, to exclude a potential dynamic bronchial compression [28]. Reference values of SVC would also be interesting in this specific high-level sportsmen population. Furthermore, spirometric values and equations were not applicable to female high-level cyclists and to different ethnic groups because of different morphological characteristics [4,5]. In the future, a collaboration to obtain a French and European database of lung function could be of great interest in order to include more high-level cyclists, women and other ethnic groups. The statistical model of Cole et al., could be used to establish new predictive values [29].

## 5. Conclusions

Cyclists have exceptional spirometric values of FEV_1_, FVC and PEF predicted by body height and suggesting a specific stimulating role of endurance exercises, probably associated with a genetic component. Therefore, the usual theoretical values should not be used so as to avoid misclassification of this high-level cycling population and misdiagnoses during individual clinical evaluations. A multicenter study could be of great interest in order to obtain more data. Due to the cycling specificity, there is a need for further investigations examining cumulative effects of the duration, severity and intensity of training and competition from adolescence to adulthood with an assessment of respiratory muscle strength and analysis of specific genetic influences.

## Figures and Tables

**Table 1:** Lung function data of professional high-level cyclists (*n* = 145).

	Mean ± SD	Minimum	Maximum
FEV_1_ (L/s) Th (%)	5.14 ± 0.69 116.8 ± 14.9	3.75 84.6	7.01 159.1
FVC (L) Th (%)	6.11 ± 0.76 116.8 ± 13.1	4.21 87.9	8.04 163
FEV_1_/FVC (%)	84.1 ± 5.9	69	96.4
PEF (L/s) Th (%)	11.68 ± 1.56 119.3 ± 16.9	8.12 88	15.8 168.7
FEF75 (L/s) Th (%)	9.35 ± 1.78 112 ± 22.4	4.80 56	14.64 159
FEF50 (L/s) Th (%)	5.95 ± 1.42 107.3 ± 25.8	2.93 51.7	9.79 175.7
FEF25 (L/s) Th (%)	2.69 ± 0.82 102.4 ± 30.4	1.23 46	5.67 230
MMEF 25–75 (L/s) Th (%)	5.35 ± 1.34 107.5 ± 26.8	2.63 51.6	9.68 188

Abbreviations: FEV_1_: forced expiratory volume in one second; FVC: forced vital capacity; PEF: peak expiratory flow; FEF75%: forced expiratory flow at 75%; MMEF: maximal midexpiratory flow between 25 and 75% of forced vital capacity; Th (%) percentage of theoretical values.

**Table 2:** Comparison of lung function between measured and theoretical values and predictive equations for the lung function (stepwise linear regression).

	Measured Values in Professional Cyclists (*n* = 145)	Theoretical Values	*p*	Equations	r	r^2^	r^2^_adj_
FEV_1_ (L/s), mean ± SD Median [5th–95th percentiles]	5.14 ± 0.69 5.11 [4.09–6.43]	4.39 ± 0.30 4.40 [3.87–4.92]	<0.001 ^a^ <0.001 ^b^	−5.48 + 0.059H	0.513	0.264	0.258
FVC (L), mean ± SD Median [5th–95th percentiles]	6.11 ± 0.76 6.12 [4.97–7.50]	5.24 ± 0.39 6.26 [4.64–5.92]	<0.001 ^a^ <0.001 ^b^	−6.9 + 0.063H + 0.025W	0.666	0.443	0.435
FEV_1_/FVC (%), mean ± SD Median [5th–95th percentiles]	84.1 ± 5.94 84.4 [71.7–92.9]	83.8 ± 1.53 84.0 [82.3–85.2]	0.51 ^a^ 0.30 ^b^	122 − 0.173H − 0.303A	0.280	0.079	0.066
PEF (L/s), mean ± SD Median [5th–95th percentiles]	11.63 ± 1.58 11.60 [9.16–14.20]	9.82 ± 0.70 9.95 [8.08–10.65]	<0.001 ^a^ <0.001 ^b^	7.08 + 0.065W	0.284	0.081	0.074
FEF75 (L/s), mean ± SD Median [5th–95th percentiles]	9.35 ± 1.78 9.35 [6.22–12.27]	8.38 ± 0.60 8.51 [6.51–9.14]	<0.001 ^a^ <0.001 ^b^	8.88 − 0.01H + 0.047W − 0.047A	0.175	0.031	0.010
FEF50 (L/s), mean ± SD Median [5th–95th percentiles]	5.95 ± 1.42 5.80 [3.69–8.71]	5.56 ± 0.38 5.59 [4.83–6.07]	<0.001 ^a^ 0.004 ^b^	8.53 + 0.017H + 0.023W − 0.053A	0.218	0.248	0.027
FEF25 (L/s), mean ± SD Median [5th–95th percentiles]	2.69 ± 0.82 2.48 [1.60–4.33]	2.63 ± 0.19 2.63 [2.32–2.95]	0.32 ^a^ 0.93 ^b^	−0.624 + 0.025H − 0.05A	0.347	0.121	0.108
MMEF 25–75 (L/s), mean ± SD Median [5th–95th percentiles]	5.35 ± 1.34 5.31 [3.25–7.30]	4.98 ± 0.27 5.03 [4.38–5.30]	<0.001 ^a^ 0.006 ^b^	6.73 − 0.058A	0.201	0.04	0.034

Abbreviations: FEV_1_: forced expiratory volume in one second; FVC: forced vital capacity; PEF: peak expiratory flow; FEF75%: forced expiratory flow at 75%; MMEF: maximal midexpiratory flow between 25 and 75% of forced vital capacity. ^a^ Paired *t*-test; ^b^ Wilcoxon rank test. A: age; W: weight; H: height; r: correlation coefficient.

## Data Availability

The data presented in this study are available on request from the corresponding author. The data are not publicly available due to ethical reasons.
